# Local action plan to promote access to the health system by indigenous Venezuelans from the Warao ethnic group in Manaus, Brazil: Analysis of the plan´s development, experiences, and impact through a mixed-methods study (2020)

**DOI:** 10.1371/journal.pone.0259189

**Published:** 2021-11-15

**Authors:** Sonia Vivian de Jezus, Adriana Ilha da Silva, Ricardo Alexandre Arcêncio, Nahari de Faria Marcos Terena, Jair dos Santos Pinheiro, Daniel Souza Sacramento, Paula de Souza Silva Freitas, Priscila Carminati Siqueira, Helaine Jacinta Salvador Mocelin, Vania Maria Silva Araújo, Rogério da Silva Lima, Thiago Nascimento do Prado, Carolina Maia Martins Sales, Ethel Leonor Noia Maciel

**Affiliations:** 1 Graduate Studies Program in Public Health, Universidade Federal do Espírito Santo, Vitória, Espírito Santo, Brazil; 2 Instituto de Ciências da Saúde, Universidade Federal de Mato Grosso, Sinop, Mato Grosso, Brazil; 3 Epidemiology Laboratory, Universidade Federal do Espírito Santo, Vitória, Espírito Santo, Brazil; 4 Escola de Enfermagem de Ribeirão Preto, Graduate Studies Program in Public Health Nursing, Universidade de São Paulo, Ribeirão Preto, São Paulo, Brazil; 5 Brazilian TB Research Network, REDE-TB, Rio de Janeiro, Brazil; 6 Department of Statistics, University of Rome La Sapienza, Rome, Italy; 7 Municipal Health Department, Manaus, Amazonas, Brazil; 8 Fundação de Medicina Tropical Dr. Heitor Vieira Dourado, Graduate Studies Program in Tropical Medicine (PhD), Universidade do Estado do Amazonas, Manaus, Amazonas, Brazil; 9 Pan-American Health Organization, Brasília, Distrito Federal, Brazil; The University of Georgia, UNITED STATES

## Abstract

**Background:**

The provision of care and monitoring of health are essential for indigenous Venezuelans from the Warao ethnic group, who are at risk of decimation.

**Objective:**

Analyze a Local Action Plan (LAP) to promote access to the health system of indigenous Venezuelans from the Warao ethnic group (IVWEG) in Manaus, Brazil.

**Method:**

A mixed-methods study was performed. Quantitative data were collected to assess the provision of care and monitoring of health conditions in IVWEG through a survey that was self-completed by healthcare providers. Qualitative narrative data were collected to gain insight into IVWEG that seek care. We applied descriptive statistics, grouping analysis (GA) by hierarchical levels, and multiple correspondence analysis (MCA). Content analysis was applied to qualitative data.

**Results:**

106 healthcare providers participated in the study, with the following characteristics: 94 (88.7%) females, 67 (63.2%) pardo race/color, 40 (37.7%) working in primary healthcare, and 49 (46.2%) nurses. In addition, 43 (40.6%) of the healthcare providers reported providing care to IVWEG. Among the providers, 89 (84%) had received training for assisting IVWEG. Additionally, 30 IVWEG were enrolled for interviews in the qualitative phase. The barriers to seeking care were language, distance to health units, and lack of money for transportation. The LAP proved to facilitate access to the health system by indigenous Venezuelans from the Warao ethnic group in Manaus. The study contributed to knowledge on a LAP addressed to IVWEG and helped improved their access to the health system, providing appropriate training for healthcare providers and other relevant actors by implementing a coherent and consistent public health policy at the local level.

## Introduction

To achieve the objectives of the 2030 Agenda for Sustainable Development and to leave no one behind, as well as the health-related commitments outlined in the New York Declaration for Refugees and Migrants (NYDRM), it is mandatory for all countries across the world that the health needs of refugees and migrants are properly addressed for achieving their safety, welfare, human dignity, and regular migration according to the countries´ ethical responsibility [1]. It is truly important to countries to ensure the implementation and monitoring of the NYDRM, as part of a humanitarian global project mainly through the strategy of universal health coverage (UHC) and solidarity and humanitarian assistance [1].

Although the percentage of the total world population who are international migrants has remained stable at about 3% for the past 60 years, global migration has shown an increasing trend in absolute numbers, mainly due to conflicts, persecution, environmental degradation and change, and lack of human security and opportunity [1]. In current years, international migration has also become a concern in the Americas [2,3], due to the civil crisis and conflicts installed in Venezuela.

According to estimates, more than five million Venezuelans had left their home country by 2020, and 264,157 had entered Brazil, of whom 101,636 had applied for refugee status and 150,196 had received authorization for temporary and/or permanent residence. Of these, about five thousand are indigenous Venezuelans people belonging to the Warao ethnic group (IVWEG), besides the Pemón, Eñepa, Kariña, and Wayúu ethnic groups, living in Brazil as legal refugees or applicants for refugee status [4].

In the area of health rights for international migrants in Brazil, the challenge for cities that receive the migrants, in accordance with the NYDRM, is to ensure access to public healthcare through the Unified Health System (SUS), promoting humanitarian assistance and encouraging the use of primary healthcare (PHC), as provided by Brazil´s 1988 Federal Constitution and the Migration Law that rules on migrants´ rights and duties and ensures access to health for all individuals residing in the country’s territory [5,6].

Because of the increase in migrants from Venezuela in Brazil, the Brazilian authorities have been encouraged to adopt more effective and broader measures in health promotion and care for these migrants in terms of surveillance, avoiding the spread of diseases and ensuring care for sick individuals [7].

In this context, Manaus, which faced problems due to the intense migratory flow, amidst the chaos and the measures imposed by the Office of the Public Prosecutor, prepared the first healthcare plan for migrants in the country with strategies to deal with health problems in PHC and a focus on IVWEG in living on the streets or in municipal shelters [8], as this population had already suffered a long process of violation of their human rights and negligence on the part of their home country, finding themselves in a situation of extreme vulnerability [7].

In addition, this ethnic group has been nearly decimated by HIV, most of the men have died, and the women who have survived are isolated, suffering from stigma, prejudice, and great difficulty/barriers in accessing health services [9]. Actions by the Local Plan have provided opportunities for access to health services and ensured the survival of this population, avoiding their extinction.

Despite numerous reports that the IVWEG are threatened with extinction [9,10], few studies have been conducted to understand the issue [11], mainly regarding the LAP and its impact through mitigating or alleviating the suffering and injustice due to the migratory process and exclusion. Here, we aimed to analyze the LAP to promote access to the health system for indigenous Venezuelans from the Warao ethnic group in Manaus, Brazil, its implementation, experiences, and impact from the perspective of the healthcare providers and the migrants themselves.

## Methods

### Study design

This is a mixed-methods study with a convergent parallel design, with a descriptive cross-sectional stage and an exploratory descriptive qualitative stage [12]. For the quantitative stage, we considered data obtained through a structured questionnaire with Healthcare Providers (HP), and in the qualitative stage, we conducted interviews addressing open questions to IVWEG.

### Study site

The study was conducted in Manaus, state capital of Amazonas (AM), Brazil. The city has 217 Basic Health Units (*Unidades Básicas de Saúde*, UBS), with approximately 46.30% coverage of primary healthcare [5]. The population of Manaus is nearly 1,832,426, as the seventh largest city in Brazil. Several islands, archipelagos, and ecological areas are found near the city, especially the Anavilhanas Archipelago, located near Novo Airão, and the Encontro das Águas (“Meeting of the Waters”), the city´s picture postcard. According to the United Nations Development Program (UNDP), Manaus has the highest human development index at 737. The component indices are: longevity, 0.826; income, 0.738, and education, 0.658 [13].

### Study population and sample

Since this is a mixed-methods study, we used two approaches in the sampling process. Probabilistic sampling was used for the quantitative phase and convenience sampling for the qualitative phase. The study population for the quantitative phase was HP who were working in Manaus, where used simple random sampling, considering a tolerable error of 5%, 95% confidence (Z_C_ 1.96), and 50% variance, assuming 10% losses [14], for a minimum sample of 88 healthcare providers.

For the qualitative stage, using convenience sampling, we considered 30 IVWEG. We chose this population because, according to the literature, they experience more difficulty in accessing health services, including inequities, discrimination, and prejudice [9].

Sex between young Warao men is common, especially before they are married, which can be a major factor for HIV transmission [10]. According to the latest estimate, around 35% tested positive for HIV [11]. There were no preventive programs addressed to this population, placing them under severe threat, especially since the political crisis in Venezuela and the breakdown of the country´s health system [10].

Many Warao are illiterate and do not speak fluent Spanish, hindering them from obtaining knowledge about the main diseases and their prevention, thus increasing the risk of extinction of this indigenous population [9]. Manaus has sheltered many migrants from Venezuela, with priority attention to IVWEG, and it was mandatory for the local government to develop a LAP addressed to them.

For defining this sample, we considered the concept of theoretical saturation adjusted to quality, transparency, and an inductive approach, meaning that data needs to be large enough to capture a range of experiences but not so large as to be repetitious [15,16]. The exclusion criteria for this phase were individuals under 18 years of age who were not living on their own or were members of the same nuclear family as a previously selected individual.

### Questionnaires and measures

We applied different tools according to the study phase. The quantitative phase used a validated and pre-tested questionnaire in Portuguese. This questionnaire was developed according to WHO guidelines that contain the main instructions for development of an Action Plan for migrants and refugees [1]. It was structured in three parts, the first consisting of participants’ sociodemographic characteristics (age, gender, and race/color); The second part contained information regarding healthcare providers who receive migrants (type of facility where they worked, occupation, number of years working in this position), and the third part contained information on the LAP addressed to IVWEG (whether activities were performed to support the migrants and improve their access to the health system, resources for the activity, previous experience with TB treatment among migrants, HIV or other health conditions, and case tracing of latent TB infection among vulnerable groups, including those with HIV at the facility where they worked, assistance for migrants at the health service, difficulties with migrants in terms of language and culture, among other factors). A copy of the questionnaire in Portuguese and English has been attached as supporting information (supplementary material 1).

In the qualitative stage, in which IVWEG were enrolled, we conducted interviews to collect data on the subjects´ own perceptions of their reality and their representations and experiences in seeking care [17]. An interview guide was employed (supplementary material 2), validated and pre-tested, with open questions and exploratory topics. The senior researcher left the migrants free to approach anything else that they considered appropriate.

The questions and points explored were related to their decision to migrate to Brazil (report and support received), life in Venezuela (social life, housing, income, employment, health conditions, structure, and social support), life after arrival in Brazil and daily life (time since entering the country, where they entered, cities they had lived in, employment, personal and family needs, immigration status, personal documents, housing conditions, health conditions, access to the service health, social support, support network, social and affective relationships) and expectations for the future.

### Data collection and procedures

Quantitative and qualitative data were collected simultaneously in September and October 2020. In both stages of the study, the field supervisors received prior training from the study`s senior researchers and assistants. This training was important for uniformity in the approach to participants, reducing bias as well as ensuring that the staff were aware of the methods applied in the study and the reasons for their application.

For the qualitative stage, a researcher with extensive experience in qualitative research conducted the interviews with the IVWEG.

In the quantitative stage, the identification of all healthcare providers was done by the Under-Secretariat of Health Management and the staff from the Municipal TB Control Program (PCT/SEMSA). A study´s survey form was sent by online link considering the criteria and eligible participants. Participants were included consecutively. In this stage, data collection used the Research Electronic Data Capture (REDCAP^®^) system through a link containing the online survey sent jointly with the informed consent form. The healthcare providers were included successively as they accessed the questionnaire, their participation was voluntary, and the questionnaire was self-administered.

Regarding the study phase with IVWEG, recruitment was facilitated by a staff worker at the shelter, who translated from Portuguese to the Warao language and introduced the study protocol, identified individuals that were interested in participating, and recorded their names on a list for subsequent contact by the researchers.

## Analytical plan

### Quantitative data: Healthcare providers

This phase initially applied descriptive statistics calculating absolute and relative frequencies for all categorical variables, allowing identification of the experiences and implementation of LAP. We also applied multivariate analysis, consisting of grouping analysis (GA) by hierarchical levels and multiple correspondence analysis (MCA). Based on similarity between the observations, the groupings aim at maximum internal homogeneity and there should be distinctions between the groups [18]. There were different profiles among the HP, and the study aimed to identify significant associations between groups on the experiences in terms of migrants´ difficulties in accessing care and to determine whether they were familiar with the LAP, with responses on their role in health promotion for IVWEG and whether they had already provided care for these people. Unanswered questions were tabulated because we considered it important to understand and highlight a healthcare provider´s choice to refrain from answering a question on the LAP. Besides, an adjusted Rand index (ARI) allowed us to validate the amounts of clusters of observations. The ARI varies from 0 to 1, where high values indicate high similarity between organization of the groups and partitions [19].

Gower´s distance was used for analysis in the quantitative and qualitative methods [20].

$$Sij=\frac{\sum_{k=1}^{p}Wijk.Sijk}{\sum_{K=1}^{p}Wijk}$$

Where K is the number of variables (k = 1, 2,…; p = total number of characteristics assessed); i and j, of any two individuals; Wijk is a weight assigned to a comparison ijk, attributing a value of 1 to valid comparisons and 0 to invalid comparisons (when the variable´s value is absent in one or both individuals); Sijk is the contribution by variable k to the similarity between individuals i and j, with values between 0 and 1. For a nominal variable, if the value of variable k is the same for both individuals, i and j, then Sijk = 1, otherwise it is equal to 0; for a continuous variable Sijk = 1 - | xik–xjk | / Rk in which xik and xjk are the values for variable k for individuals i and j, respectively, and Rk is the range of variation for variable k in the sample. Division by Rk eliminates the differences between the variables´ scales, producing a value within the interval [0, 1] and equal weights.

The next stage consisted of analyzing the similarity in the respective variables, that is, two categories are similar when the choices by different individuals coincide.

### Qualitative data: IVWEG

To analyze IVWEG’s perspectives on the LAP, all the interviews were transcribed in full and manually analyzed, after in-depth reading of the empirical data. Data from the empirical material were analyzed using content analysis [21], and the following stages were adopted:

*Appropriation of the textual corpus* (adequate classificatory plan), inference (when one logically deduces something from the target content), description (enumeration of the text´s characteristics, summarized after analytical treatment), and interpretation (significance assigned to the characteristics enumerated during description) [22].

The data´s results and discussion were organized in empirical analytical categories that emerged from the data and were not defined in advance. The categories were grouped in *barriers and hindrances*: language, mobility/travel, socioeconomic conditions and sustainability, lifestyle and culture, case-resolution capacity, inequity in health services access, housing conditions; and *facilitators*: implementation of a LAP for *IVWEG* for supporting institutions and healthcare providers, documentation (especially the Unified Health System-SUS card), and organization/ coverage of social health services and benefits.

#### Ethical approval

The study protocol was approved by the Institutional Review Board of Universidade Federal do Espírito Santo, National Research Ethics Commission (CONEP) (review no. 3.953.347), and PAHOERC (no. 0204.03). All participants who agreed to participate in the study provided written informed consent. Participation in this study was entirely voluntary, and participants could withdraw from the study at any time if they wished.

## Results

### Quantitative study: Healthcare providers´ perspectives

Table 1 lists the main characteristics of the 106 healthcare providers who participated in the study: 94 (88.7%) females, 67 (63.2%) pardo race/color, and 40 (37.7%) working in a Basic Healthcare Unit. The largest professional category was nurses 49 (46.2%), followed by nurse technicians 25 (23.6%).

**Table 1:** Characteristics of the healthcare providers who participated in the study in Manaus, Brazil (2020).

Variables	n	%	% Ac.
**Gender**			
Female	94	88.7%	88.7%
Male	12	11.3%	100%
**Race/color/ethnicity**			
Pardo	67	63.2%	63.2%
White	30	28.3%	91.5%
Black	6	5.7%	97.2%
Missing	1	0.9%	98.1%
Asian-descendant	1	0.9%	99.1%
Criollo	1	0.9%	100.0%
**Type of health unit**			
UBS	40	37.7%	37.7%
UBSF	25	23.6%	61.3%
Other	24	22.6%	84.0%
ESF in UBS	10	9.4%	93.4%
Polyclinic	6	5.7%	99.1%
Referral outpatient clinic	1	0.9%	100.0%
**Profession**			
Nurse	49	46.2%	46.2%
Nurse technician	25	23.6%	69.8%
Other	17	16.0%	85.8%
Physician	12	11.3%	97.2%
Nurse assistant	3	2.8%	100.0%

UBS = Basic Health Service; UBSF = Basic Family Health Service; ESF = Family Health Strategy.

Table 2 list aspects of the organizational structure for reception of IVWEG and their access to healthcare units in the health system in Manaus. Some 43 (40.6%) of the healthcare providers had assisted them, and 64 (60,4%) reported that this activity had been performed for more than two years. However, most of the healthcare providers (63; 59.4%) were unaware of the specific resources for this activity. In addition, 93 (87.7%) have assisted IVWEG, and 54 (50.9%) stated that there were no barriers for IVWEG to access the health units, while 44 (41.5%) reported that there were such obstacles.

**Table 2:** Organizational structure of the health facilities for receiving IVWEG after launching of the LAP in Manaus, Brazil (2020).

Variables	n	%	% Ac.
**Provides care for IVWEG**			
No	49	46.2%	46.2%
Yes	43	40.6%	86.8%
Don´t know	9	8.5%	95.3%
Missing	5	4.7%	100,0%
**Time the health unit has provided care for IVWEG**			
More than 2 years	64	60.4%	60.4%
From 1 to 2 years	18	17.0%	77.4%
From 6 months to 1 year	8	7.5%	84.9%
Don´t know	8	7.5%	92.5%
Less than 6 months	5	4.7%	97.2%
Did not answer	3	2.8%	100.0%
**Resources for providing care to IVWEG**			
Don´t know	63	59.4%	59.4%
No	20	18.9%	78.3%
Yes	18	17.0%	95.3%
Did not answer	5	4.7%	100.0%
**Works at unit that provides care for IVWEG**			
Yes	93	87.7%	87.7%
No	7	6.6%	94.3¨%
Don´t know	6	5.7%	100.0%
**Time since first care provided to IVWEG**			
More than 2 years	34	32.1%	32.1%
From 1 to 2 years	25	23.6%	55.7%
From 6 months to 1 year	17	16.0%	71.7%
Less than 6 months	13	12.3%	84.0%
Don´t remember	11	10.4%	94.3%
Did not answer	6	5.7%	100.0%
**Presence of IVWEG**			
Yes	56	52.8%	52.8%
No	25	23.6%	76.4%
Don´t know	17	16.0%	92.5%
Don´t remember	7	6.6%	99.1%
Did not answer	1	0.9%	100.0%
**Barriers to care for IVWEG**			
No	54	50.9%	50.9%
Yes	44	41.5%	92.5%
Don´t know	6	5.7%	98.1%
Did not answer	2	1.9%	100.0%

As for training of human resources to assist IVWEG in the city of Manaus, Table 3 shows that 89 (84%) of the healthcare providers had received training, 82 (77.4%) were aware of the Action Plan, 88 (83%) reported that the LAP had been publicized by the Municipal Health Secretariat, and 88 (83%) acknowledged the Action Plan´s importance.

**Table 3:** Implementation of the LAP addressed to IVWEG and healthcare providers´ assessment of the Plan´s importance in Manaus, Brazil (2020).

Variables	n	%	% Ac.
**Has received training to provide care for IVWEG**.			
Yes	89	84.0%	84.0%
No	12	11.3%	95.3%
Did not answer	5	4.7%	100.0%
**Awareness of LAP for IVWEG**			
Yes	82	77.4%	77.4%
No	18	17.0%	94.3%
Did not answer	5	4.7%	99.1%
Don´t know	1	0.9%	100.0%
**Communication of LAP for IVWEG**			
Communication by Health Secretariat	88	83.0%	83.0%
Communication by Health Unit Manager	8	7.5%	90.6%
Coworker	6	5.7%	96.2%
Did not answer	4	3.8%	100.0%
**Agree with the Plan´s importance for adequate management of population of IVWEG**			
Agree	88	83.0%	83.0%
Agree totally	9	8.5%	91.5%
Neither agree nor disagree	8	7.5%	99.1%
Did not answer	1	0.9%	100.0%
**Would recommend the Action Plan for IVWEG to other cities that receive these migrants**			
Recommend in full	88	83.0%	83.0%
Recommend parts of Action Plan	13	12.3%	95.3%
Don´t know	4	3.8%	99.1%
Did not answer	1	0.9%	100.0%
**Agree that it is the Health Secretariat´s responsibility to promote the health of IVWEG**.			
Agree	60	56.6%	56.6%
Agree totally	19	17.9%	74.5%
Neither agree nor disagree	14	13.2%	87.7%
Disagree	9	8.5%	96.2%
Disagree totally	3	2.8%	99.1%
Did not answer	1	0.9%	100.0%

In the survey, 88 (83%) of healthcare providers recommended the Action Plan´s expansion to other municipalities that receive IVWEG, and 19 (17.9%) totally agreed that it is the responsibility of the Municipal Health Secretariat to establish protocols and policies to receive this population.

**Fig 1:**
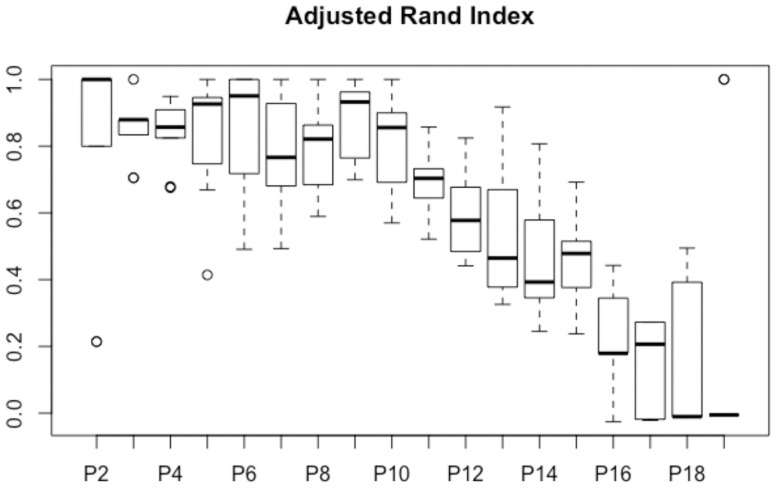
Adjusted Rand index (ARI) applied to validate the amounts of clusters regarding the healthcare providers and implementation of the LAP in Manaus, Brazil (2020).

The optimal number of clusters was five profiles of respondents. Fig 1 shows the hierarchical grouping´s dendrogram and maps the questions that significantly distinguished individuals from each other.

**Fig 2:**
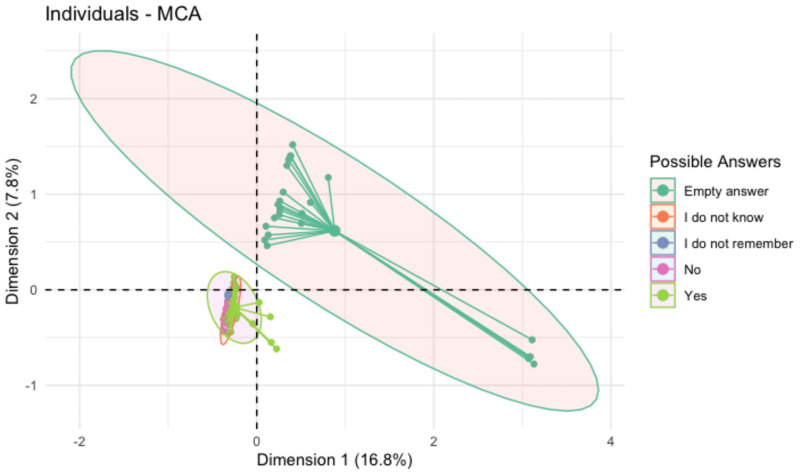
Multiple correspondence analysis regarding difficulties for healthcare providers to promote the care for IVWEG in health units in Manaus, Brazil (2020).

In Fig 2, the adjusted Rand index corroborated the optimal number of clusters, since the values of the 5 partitions are close to 1 and the variability is less than other quantities.

Table 4 describes the analysis and hierarchization of groups by the characteristics of healthcare providers who were responsible for implementation of the Local Action Plan, where the concentrations occurred as follows. The age brackets and gender distribution in the groups were similar. The types of health units were characterized by white race/color profile, contact for longer than two years with the Action Plan, and the care itself. Another salient point was group 1, referring to the healthcare workers´ profession. In group 3, the type of unit was the Basic Family Health Unit, which does not provide care in the Local Action Plan. In group 2, there was a relevant share of healthcare providers who opted not to answer some questions on the work performed. Hierarchical level cluster analysis was applied to the results presented in Table 4.

**Table 4:** Analysis and hierarchization of groups by the characteristics of healthcare providers who were responsible for implementation of the LAP to promote access to the health system by IVWEG in Manaus, Brazil (2020).

	Cluster 1	Cluster 2	Cluster 3
**Age**:	43	46	34
**Gender**:	Female	Female	Female
**Color**:	White	Pardo	Pardo
**Type of health unit**:	Other	UBSF	UBS
**Occupation**:	Nurse	Nurse technician	Nurse
**Years worked**:	5	14	10
**Performs activities in Action Plan for Health of IVWEG?**	Yes	No	No
**Unit performs diagnosis of TB, HIV, and other diseases?**	Yes	Yes	Yes
**Unit provides treatment of TB, HIV, and other diseases?**	Yes	Yes	Yes
**Health unit provides care for IVWEG?**	Yes	No	Yes
**Have you ever cared for an IVWEG in your routine work?**	Yes	No	Yes
**When did you provide care for IVWEG for the first time?**	More than 2 years ago	-	Between 1 and 2 years
**Was any of the IVWEG?**	Yes	-	No
**Have you ever provided care for an IVWEG for TB, HIV, and diagnosis or treatment of other diseases?**	Yes	-	No
**Do you see any barrier/difficulty to caring for IVWEG in health units in Manaus?**	No	Yes	No
**Have you received training to provide care for Venezuelan migrants?**	No	No	No
**Would you like to receive training to provide care for IVWEG?**	Yes	Yes	Yes
**Are you familiar with the Action Plan for Health of IVWEG?**	No	No	No
**In relation to the statement: It is the role of the Health Secretariat to promote the health of IVWEG**.	Agree	Agree	Agree
**It is the role of the healthcare worker in the SUS to promote the health of IVWEG. in Manaus**:	Agree	Agree	Agree

An adjusted Rand index was used to validate the amounts of clusters obtained according to the healthcare providers’ profile, as shown in Fig 1.

Multiple correspondence analysis (MCA) of the individuals and variables was performed, allowing identification of the main characteristics of the healthcare providers who have implemented the LAP as well their assessment of the Plan. Fig 2 shows the answers from healthcare providers regarding difficulties for providing the care to IVWEG in the health units. Based on this analysis, we observed different patterns of answers: missing, did not know, did not remember, yes or no. The MCA of the individuals informed approximately 25% of the variability in the data on care for IVWEG. The group that stood out from the whole was the one that opted not to answer, while those that responded had similar variability. However, those who had not provided care to IVWEG tended not to answer other questions.

**Fig 3:**
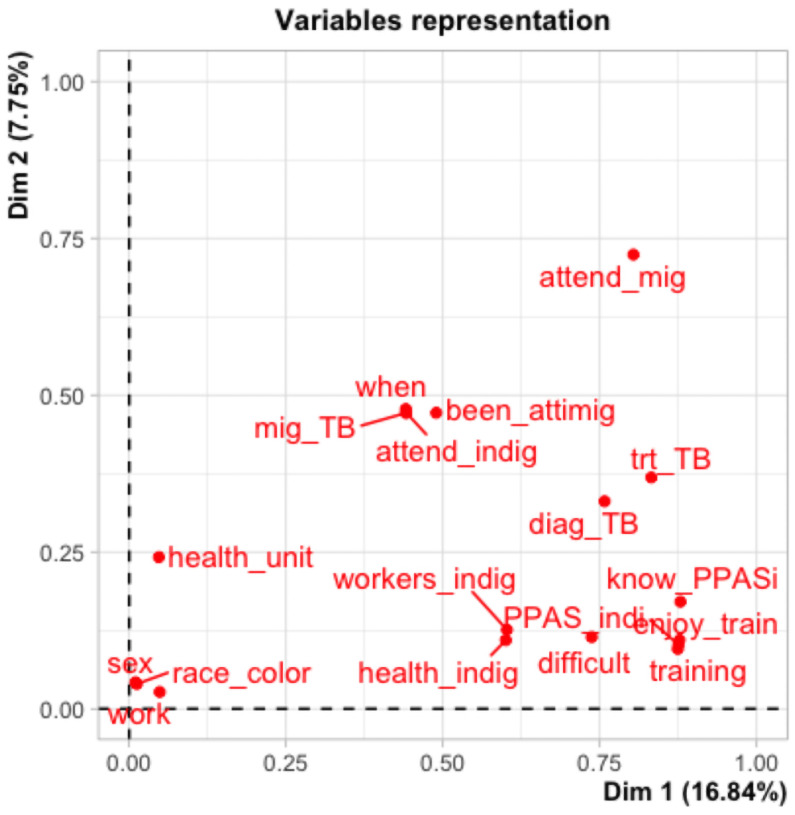
Perceptual map of the categorical variables associated with implementation of the LAP for promoting access to the health system by IVWEG in Manaus, Brazil (2020).

Fig 3 presents the main healthcare providers´ characteristics. The perceptual map of the categorical variables (Fig 3) revealed questions with proximity and similarity, such as “SUS_indi” and “prof_indi”, which are statements on health promotion for IVWEG, while another question pertained to care for them, while race/color, and type of health unit, not analogous to other questions.

### Qualitative study: From the perspective of IVWEG

Thirty interviews were conducted. Table 5 lists the quotes by participants according to categories and subcategories.

**Table 5:** IVWEG´ perceptions of barriers to access to the health system and facilitators with the LAP in Manaus, Brazil (2020).

Classification and subclassification		Narratives
**Barriers and hindrances**	**Language**	*“[…] When I don´t understand what the doctor says*, *I ask what the important part is*, *and when*…*the doctor or nurse explains*, *I pass it on to my wife*, *about the importance of the child receiving that kind of care or vaccine*, *so they can give it” (Interviewee W3)*. *“[…]“I broke my nose in a family fight and I went to the doctor and they didn´t say anything*, *they just took an x-ray and I couldn´t communicate or talk with the doctor” (Interviewee W11)*. *“My daughter*, *a year and two months old*, *got sick with diarrhea and vomiting*. *I went to the hospital*, *and nobody could understand when I spoke*. *I came back to the shelter to get a neighbor lady who spoke the language [Portuguese]*. *So*, *my daughter was admitted with her mother*, *and I went to see what the girl had*. *I believe my child died because the doctor couldn’t understand me” (Interviewee W11)*. *“The care and the health service are good*. *The only thing is that when we get sick*, *it´s difficult to speak and explain*. *The people in charge of the shelter don´t understand too well*.*” (Interviewee W17)*
	**Mobility and transportation**	*“[…] When my wife felt the pain to have the baby at four in the morning*, *I got up*, *took a shower*, *there was a telephone*, *I called Uber*, *and I went to get her myself” (Interviewee W3)*. *“Here in this shelter where I am*, *it´s far*. *I´ve used the health service*, *the service is good*, *but here in this shelter*, *to use the clinic it´s too far*, *because you have to go on foot*.*” (Interviewee W8)*. *“[…] I asked to enter the terminals without paying*, *to be able to take a bus*, *because it was far from where I could find medication (Interviewee W1)* *“Here in the shelter where I am is far*. *(Interviewee W8)*.
	**Socioeconomic conditions and sustainability**	*“Access here is difficult*. *I´m a chief*, *and I speak with the committee*, *and they give me a doctor´s prescription for example and ask me to get that medicine*. *So*, *with no money for the fare*, *many times I´ve gone on foot to get the medicine for another indigenous person that was sick*. *With no kind of expenses paid*, *I´ve already gone hungry*, *I went on foot*, *I asked to enter the terminals to take a bus because it was too far to where I could find the medication requested by the city government staff that care for the shelter” (Interviewee W1)*. *“[…] I´ve used the health service*, *I received good care*, *but here in the current shelter the clinic is far away*, *and we have to go on foot” (Interviewee W8)*. *“The treatment was good*, *but I had to purchase the medication after my surgery*. *I couldn´t get it free” (Interviewee* W6). *“With the pandemic*, *there were changes in the shelters*. *In the process*, *my four-year-old daughter was diagnosed with tuberculosis and was hospitalized […] I also tested positive*, *my PPD was positive*, *and I didn´t receive treatment because the closest clinic didn´t have the medication*, *and my four-year-old daughter was still hospitalized” (Interviewee W9)*.
	**Lifestyle and culture**	*One of the principles for maintaining health is a good diet*, *which is not happening*. *We would like to cook our own meals … We already spoke to the coordinators*, *but apparently nobody listened*, *because they didn´t find a solution” (Interviewee W4)*. *“[…] Many of them believe that they are spiritual diseases or diseases from witchcraft*, *and that if they go to the hospital*, *they end up dying*, *because they use medicine that they shouldn´t*. *There have already been cases where this happened*, *so they´re afraid to go*, *and I´m afraid*, *too […]” (Interviewee W15)* *“*…*the food that comes to the shelter is not food from the Warao culture*, *and oftentimes I don´t eat it” (Interviewee W21)*
	**Case resolution**	*“I went to the doctor because my son had a toothache*. *The doctor referred him to have the tooth pulled out*. *And it´s been five months*, *and no answer about when he´s going to be treated*, *and the tooth is already poking out of the gums*. *And he´s not eating anymore because of the toothache” (Interviewee W18)*. *“My wife is pregnant*, *and I´m unhappy with the care*. *Why haven´t they done an ultrasound*? *And the children aren´t being seen or monitored daily like it was in the last shelter” (Interviewee W4)*. *“The health issue is going badly*, *because I think there should be medical and nursing care at the shelter” (Interviewee W2)*.
	**Environmental and housing conditions**	*“The conditions are not good at the shelter*. *I lived at a shelter [*…*] for a year*, *and I believe my children caught tuberculosis there*. *I never imagined that could happen” (Interviewee* W7).
	**Support institutions**	*“ADRA had the children vaccinated and took them to the hospitals*. *ADRA made a point of taking pregnant women and children to see the doctor*, *for medical care*.*” (Interviewee W3)*. *“When ADRA* [Adventist Development and Relief Agency] *came to the Alfredo Nascimento Shelter*, *the health situation inside the shelter cleared up*, *because they started to hold meetings and talks about HIV*, *about women*, *about TB” (Interviewee W9)*.
**Facilitators**	**Impact of LAP regarding the coverage by SUS**	*“I received my SUS card here at the Alfredo Nascimento Shelter*, *it was quick*, *and I got it myself*, *I had no problem getting it*.*” (Interviewee W3*
	**Social health services ensured by Local Action Plan**	“*I never had any problem with the health service*, *just the ambulance” (Interviewee* W1). *“I was 8 months pregnant when I came to Brazil*. *The doctor oriented me*, *referred me for care*, *I received diapers*, *baby clothes*, *and I participated in group discussions about breastfeeding” (Interviewee W5)*. *“When I need care*, *it´s scheduled by a caregiver*. *and they tell me the day*, *time*, *and place I´m supposed to go*, *and I´m always treated very well”*. *(Interviewee W5)*. *“I´ve never been sick*, *but I´ve already used the medical care*, *I´ve used the nursing care*, *and I have nothing to complain about” (Interviewee W3)*. *“When I need the health service*, *I speak to the coordinators at the shelter*, *and they schedule the appointment” (Interviewee W14)*. *“Here in the community*, *at the shelter*, *they have caregivers*, *so the caregivers ask if people are sick*, *if they´re feeling something*, *so if they´re feeling a headache*, *or fever*, *the caregivers call in a nurse or doctor to come here to the shelter to take a look*, *to do the treatment” (Interviewee W21)* *“About the TB diagnosis… First*, *they discovered it in my six-year-old niece*, *and she was cured*. *Then my mother caught it*, *and she was hospitalized for six months*, *and I later caught it*, *too*. *I stopped eating*, *got kind of sick*, *but now I´m in treatment in the SUS*.*" (Interviewee W27)*. *“Here in Brazil*, *they have medication*, *besides the appointment*. *It´s been a lot better here because you can respond to the prescribed treatment*.*” (Interviewee W8)* *“There´s a health committee in the shelter*. *When one of my children gets sick*, *I tell the health committee*, *and the health committee tells the SEMASC* [Department of Social Services, Manaus] *team*, *which schedules the appointment or even takes the child to the doctor if necessary*.*” (Interviewee W28)*

The analysis of perceptions on the provision of care in the health system in Manaus for IVWEG revealed the following barriers: language for communication; distance from the shelters to the healthcare units; lack of work and income to purchase medicines and sustain treatment; Warao cultural beliefs in relation to access to health services, since they interpret diseases as witchcraft or spiritual problems; lack of case resolution in procedures and tests; inequities in healthcare; and conditions in the shelters that hinder TB prevention and treatment.

Participants´ comments showed that the LAP was a facilitator, proving to be an important strategy to promote access to the health system of by indigenous Venezuelans from the Warao ethnic group in Manaus. They cited the following relevant actions: staff mediation for vaccination of Warao children, follow-up in the hospitals, acquisition of the SUS card, and educational talks on diseases and breastfeeding.

## Discussion

The study showed that the LAP positively impacted access by IVWEG to the healthcare system, which contributed to improving their health conditions and promoting their integration to the city´s main services, including those related to social benefits. IVWEG were the most vulnerable members among the Venezuelan migrants that have come to Manaus.

Healthcare providers recognized the importance of the Local Plan addressed to IVWEG and reported having been trained to provide care to IVWEG. They also reported experiences with detection of diseases such as TB, HIV, diabetes, and others. Vaccination coverage, mainly in children, was another benefit from the Plan. From the perspective of the IVWEG, the participants cited numerous difficulties in the adaptation process, including prejudice, stigma, and the language barrier, mainly because they spoke their own Warao language, which the local population in Manaus did not understand.

The hierarchical grouping dendrogram and perceptual maps generated with the MCA revealed profiles that distinguish healthcare providers from each other, identifying some groups with less difficulty including and promoting the health of IVWEG, specifically nurses, women, individuals 43 years or older, those who had worked in the unit less than 5 years, and those working in traditional units or specialized services.

Despite international conventions to protect the rights of migrants, reports in various parts of the world point to lack of access to health promotion, prevention and care, and financial protection to afford such services. Such problems were corroborated in the current study in relation to indigenous Venezuelans from the Warao ethnic group in Manaus, Brazil [1]. According to the WHO (2016), the country of origin generally has a less developed health service or disrupted health systems due to protracted crises. This contributed greatly to precarious health conditions and numerous deaths among Warao men [9]. This issue becomes particularly complex with indigenous peoples, whose habits and culture are generally different from those of healthcare providers. According to the literature, indigenous persons usually suffer from a wide range of additional barriers including experiences of discrimination and racism, making access even more difficult [23].

The data showed that most of the healthcare providers were women, nurses, with pardo color/race. Understanding the health-disease process from an anthropological and social perspective is a key component for all healthcare services and is also considered essential to the structuring of health professions in Brazil [24,25]. Reframing some processes without prejudice and stigma is essential for better performance of the services, specifically those working with indigenous peoples. IVWEG cited the presence of nurses and physicians as important for solving their health problems in the shelters and the health units, and the LAP has facilitated this process through the qualification of the action and training of these professionals.

As provided by Brazilian Law 8.080/90, the conditions for health promotion, protection, and recovery and the organization and functioning of health services are a duty of the state and are available to everyone residing in the country’s territory [6]. The Migration Law corroborates the premises of the Unified Health System (SUS) by determining that Brazilian migratory policies are ruled by principles and guidelines, including universal coverage, indivisibility, interdependence of human rights, and humanitarian solidarity [5].

Concerning migrants´ reception, 41.5% of the healthcare providers acknowledged the existence of barriers to care for this group. Most of the interviews with the IVWEG revealed language barriers, hindering the explanation of their complaints and understanding of the healthcare providers´ instructions related to illness, diagnosis, treatment, and referrals. The same situation was evidenced in a study on experiences with access to primary healthcare by African immigrants and refugees in a meta-ethnography on the experiences in maternity services in Canada, where communication was found to be a barrier to care [26,27].

It is thus extremely important to conduct training to familiarize healthcare providers with the migrants´ language and customs [28]. Such training should also address the fact that the Warao culture conceptualizes diseases as witchcraft or spiritual problems. Cultural factors were also identified as relevant among African immigrants [29].

The training received by most of the healthcare providers (84%) consisted of comprehensive care for the Venezuelan migrants. Although 77.4% of the healthcare providers stated that they were aware of the Action Plan, most agreed that there is a need for the government to promote the health of indigenous Venezuelans (56.6% agreed; 17.9% totally agreed).

The epidemiological profile of IVWEG in Brazil calls for strategies to mitigate this situation [5]. According to an evidenced-based report by UCL-Lancet on Migration and Health, limited access is exacerbated by administrative, financial, legal, and language barriers, and it is necessary to establish protocols and policies to decrease these healthcare inequities [30].

The quotes by the Warao immigrants revealed their difficulty in reaching the health services due to the distance from the shelters provided by the municipal government, besides the overcrowding in this housing. The choice of areas to house them far from the city center was due to lower costs. Reception of the Warao immigrants in Manaus has been made possible by subsidized rent and/or shelters provided by the municipal government through an agreement with various parties (Office of the Public Prosecutor, Operation Welcome, Amazonas state government, and Manaus municipal government). The excessive number of individuals in the housing is justified by attempting not to separate recently arrived nuclear families, respecting the Warao people´s cultural processes, even though this facilitated the transmission of respiratory diseases.

An alarming situation concerns the reports of advanced-stage TB and deaths in Venezuelans in shelters, even though most of the healthcare providers had already conducted TB diagnosis and treatment in this group. It is well-known that social determinants of TB should be tackled not only by strengthening programs for control, diagnosis, and treatment, but also through measures affecting the social determinants of TB by strengthening social protection and interventions to support means of subsistence [31,32]. In addition, people with no schooling, who watch little or no television, and who have no internet access show higher odds of lacking knowledge on TB [33].

Finally, it is important to highlight the Action Plan´s importance both for the city of Manaus and for other areas of Brazil, given that the particularly vulnerable conditions migrants largely determine their health status [29]. The Action Plan allows mitigating barriers and promoting action aimed at well-being, health promotion, and prevention of diseases, including early diagnosis of infectious diseases such as tuberculosis and HIV. Indigenous Venezuelans from the Warao ethnic group have their very survival threatened, because many of the men have died of AIDS. The Action Plan has thus been highly relevant for early diagnosis of communicable diseases and for avoiding new infections through strategies of popular education, with respect for their values and culture [9].

The LAP has impacted the survival of IVWEG, promoting structural changes in the scenario under study, improving the cultural, organizational, geographic, and economic access to health services [23]. The evidence shows that the Plan has provided a great opportunity to address access, because it has prepared the healthcare providers to manage the different types of social and cultural determinants of health faced by the migrants.

Unfortunately, not all Brazilian cities that have received Warao immigrants are as satisfactorily structured and organized through a strategic and relevant Plan as in Manaus, which should be recognized in the study. Despite numerous difficulties with underfunding of the Unified Health System, since Brazil is experiencing a serious political and economic crisis, with progressive reduction of financial resources for the SUS in recent years [34], the study revealed major strides in the universal system from the perspective of ethical, humanitarian, and social inclusion and equity for IVWEG.

The Plan was designed in accordance with the recommendations from the 2030 Agenda for Sustainable Development, the NYDRM, and WHO, contributing to the definition of policies and practices, with multi-sector and multidisciplinary approaches, revealing an interesting aspect to be evaluated in the scenario under study. Namely, Manaus is unique among Brazilian cities, having designed a Plan with this objective, which makes the study truly original and novel.

The study was conducted in a scenario heavily affected by the COVID-19 pandemic. However, based on the findings, we assume that LAP has avoided the spread of SARS-CoV-2 and a catastrophic event among the IVWEG.

One limitation was that the study was only conducted in the city of Manaus. However, since this was the first Brazilian municipality to develop an action plan for IVWEG, the experience may be applicable to other settings. The study´s strengths include the mixed-methods design, which allowed widely exploring the situation with IVWEG the ways the Plan has ensured their health equity. The findings from the two study phases were convergent and mutually complementary, as recommended in the literature [12]. The healthcare providers assessed the Plan from their perspective, and this assessment was confirmed by the IVWEG, providing greater completeness and consistency to the findings. The interviews with different healthcare providers and with IVWEG helped triangulate the results.

## Recommendations

The main lesson from the initiatives in the implementation of a LAP for IVWEG in the city of Manaus is the need to create a national policy for this population, with inter-sector actions involving health, social assistance, and human rights, promoting this population´s socioeconomic welfare and improving the social determinants of health in the migrants´ living conditions to prevent infectious diseases such as tuberculosis.

## Conclusion

Future studies are important for understanding how this LAP can serve as a model for other municipalities that receive migrants and how we can improve it so that migrants, whether indigenous or nonindigenous, have equal access to effective and universal healthcare, as provided by the legislation governing the Unified Health System (SUS), especially taking language and culture into account.

## Supporting information

S1 File English questionnaire health providers. (DOCX)

S2 File Semi structured script for interview with migrants. (DOCX)

S1 Dataset (CSV)
