# Supplementary material for: Local action plan to promote access to the health system by indigenous Venezuelans from the Warao ethnic group in Manaus, Brazil: Analysis of the plan´s development, experiences, and impact through a mixed-methods study (2020)
Source: PLoS One. 2021 Nov 15;16(11):e0259189. doi: 10.1371/journal.pone.0259189 (PMC8592448; doi:10.1371/journal.pone.0259189)
Supplement: S1 File — (DOCX) [file pone.0259189.s001.docx]

# English questionnaire

# Invitation, Phase 3

Dear primary healthcare worker in Manaus, Amazonas, Brazil, this questionnaire is part of a study by the Federal University of Espírito Santo and the World Health Organization, approved by the Municipal Health Department, to understand aspects of healthcare for international migrants in the city of Manaus. You are not required to answer it. We guarantee the data´s confidentiality, and all the information provided will only be analyzed collectively.

Do you want to participate in the survey?
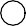
 No
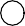
 Yes

Thank you for participating. Please click on (submit) to close the questionnaire.

Which municipality do you work in?
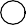
 Manaus
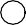
 Another municipality

Thank you for participating, but you don´t meet the requirement of working in Manaus to participate in the survey. Please click on (submit) to close the questionnaire.

Date of Interview:

(Please click on Today)

Date of birth:

(The date of birth should be completed in the day-month-year format, for example: 02-12-2000)

How old are you?

Thank you for participating, but you don´t meet the requirements (working in Manaus and 18 years of age or older) to participate in the survey. Please click on (submit) to close the questionnaire.

Since you meet the requirements (working in Manaus and age 18 years or older), you may continue participating in the survey. To continue, it´s necessary to read the free and informed consent form (FICF) and accept it if you agree to participate. To read the full text of the FICF for the Research Project on Tuberculosis and Migrants in the BRICS Countries, the Case of Brazil, access here.

FREE AND INFORMED CONSENT: I hereby declare that I have read the FICF and am aware of the use of my deidentified data in reports and analyses pertaining to the above-mentioned study. To agree or not agree to participate in the survey, click on (submit) to proceed or close the questionnaire.


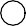
 I don´t agree to participate
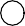
 I agree to participate

# Web Survey Phase 3

Dear primary healthcare worker in Manaus, Amazonas, Brazil, this questionnaire is part of a study by the Federal University of Espírito Santo and the World Health Organization, approved by the Municipal Health Department, to understand aspects of healthcare for international migrants in the city of Manaus. You are not required to answer it. We guarantee the data´s confidentiality, and all the information provided will only be analyzed collectively.

**Interview Data**

1. Sex


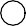
 Female
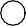
 Male

1. Race/color/ethnicity:

White
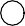
 Black
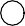
 Asian-descendant
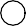
 Pardo
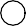
 Indigenous
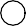
 Creole
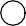
 I don´t want to answer ○Other


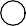

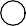


2.1- Please, write the other race/color/ethnicity that you identify with:

1. Type of healthcare unit where you work

PHC
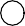
 FHS in PHC
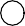
 FHPHC (Family Health Primary Care Unit)
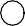
 Polyclinic Outpatient Referral Clinic
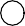
 Other


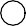

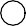


1. What is the other type of healthcare unit where you work?
2. Occupation:


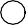
 Physician
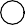
 Nurse
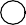
 Nurse Technician
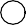
 Nurse Assistant
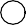
 Other

4.1- What is the other occupation?

1. Number of years that you have worked at this position:
2. Does the healthcare unit where you work conduct activities in the Action Plan for Health Promotion and Healthcare for Indigenous Venezuelans?


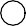
 Yes
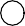
 No
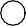
 I don´t know

6.1- When did the unit begin to conduct these activities?

Less than 6 months ago
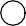
 6 months to 1 year
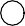
 1 to 2 years
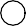
 More than 2 years I don´t know


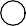

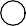


6.2- Has the unit received any resources for these activities?
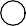
 Yes
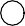
 No
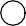
 I don´t know

6.2.1- What kind of resources?


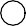
 Financial
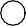
 Inputs
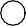
 Staff
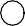
 Other
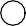
 I don´t know

6.2.2- What other kinds of resources?

6.3- Has the unit interrupted these activities any time?
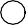
 Yes
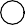
 No
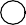
 I don´t know

6.3.1- What was the main reason?

Lack of material resources
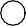
 Lack of staff
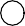
 Lack of demand
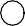
 Other I don´t know


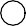

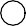


6.3.2- What other reasons?

1. Does the unit where you work conduct TB diagnosis?
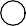
 Yes
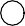
 No
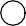
 I don´t know
2. Does the unit where you work conduct TB treatment?
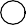
 Yes
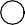
 No
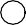
 I don´t know
3. Does the unit where you work treat international migrants?
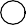
 Yes
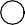
 No
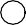
 I don´t know

9.1- Have you treated any international migrant in your routine work?
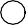
 Yes
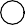
 No
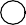
 I don´t remember

9.1.1- When was the first time you treated international migrants?

Less than 6 months ago
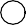
 6 months to 1 year ago
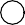
 1 to 2 years ago
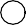
 More than 2 years I don´t remember


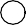

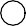


9.1.2- Among the international migrants treated in your routine, were any indigenous Venezuelans?
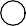
 Yes
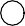
 No
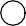
 I don´t know
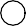
 I don´t remember

9.1.3- Have you ever rece an international migrant for TB diagnosis or treatment?
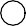
 Yes
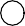
 No
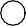
 I don´t remember

1. Do you identify any barriers or difficulties for treating Venezuelan migrants in the health units in Manaus?


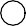
 Yes
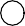
 No
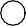
 I don´t know

10.1- Check the difficulties you identify (check as many as necessary):

Language Culture

Access/follow-up Referrals Support network Documents Other

10.2- What other difficulties?

1. Have you received any training for treating Venezuelan migrants?
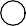
 Yes
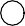
 No
2. Would you like to receive training for treating international migrants?
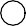
 Yes
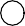
 No
3. Are you familiar with the Action Plan for Health Promotion and Healthcare for Indigenous Venezuelans in Manaus?
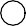
 Yes
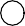
 No
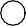
 I don´t know

13.1- How did you learn of the plan?

Communication from the Health Department
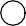
 Communication from health unit manager
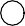
 Coworker Other


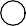

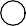


13.1.2- How else did you learn of the Plan?

13.2- In relation to the statement, “The Action Plan for Health Promotion and Healthcare for Indigenous Venezuelans in Manaus helps the health situation of this population”:

I agree completely
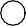
 Agree
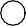
 Neither agree nor disagree
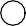
 Disagree Disagree completely


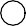

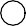


13.3- Would you recommend the implementation of similar plans in other cities?
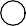
 I recommend fully
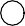
 I recommend in part
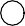
 I don´t know
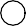
 I don´t recommend

13.4- If you could suggest any activity within the Action Plan for Health Promotion and Healthcare for Indigenous Venezuelans in Manaus, what would it be?

1. In relation to the statement, “It is the role of the Health Department to promote the health of indigenous Venezuelans in Manaus”:


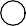

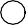


I agree completely Agree Neither agree nor disagree Disagree Disagree completely

1. In relation to the statement, “It is the role of healthcare professionals in the Unified Health System to promote the health of indigenous Venezuelans in Manaus”

I agree completely Agree Neither agree nor disagree Disagree Disagree completely

Thank you for reaching the end of the survey. Please, click on (submit).

**Questionário em Português**

**Convite, Fase 3**

Prezado(a) profissional da Atenção Primária de Manaus, este questionário faz parte de um estudo da Universidade Federal do Espírito Santo e Organização Mundial de Saúde, com aprovação da Secretaria Municipal de Saúde, para entender aspectos da atenção à saúde de migrantes internacionais na cidade. Você não precisará se identificar para respondê-lo. Garantimos o sigilo dos dados e todas as informações fornecidas serão apenas analisadas em conjunto.

Você quer participar da pesquisa?

Não Sim

Obrigada pela sua participação. Por favor, clicar em (submit) para encerrar o questionário.

Qual município você trabalha?

Manaus Outro munícipio

Obrigada pela sua participação, mas você não tem o requisito de trabalhar em Manaus necessário para participar da pesquisa. Por favor, clicar em (submit) para encerrar o questionário.

Data da Entrevista:

__________________________________

(Por favor, clicar no botão Hoje.)

Data de nascimento:

__________________________________

(A data de nascimento deve ser digitada no formato de dia-mês-ano, por exemplo: 02-02-2000) A sua idade é:

__________________________________

Obrigada pela sua participação, mas você não tem os requisitos (trabalhar em Manaus e ser maior de 18 anos) necessários para participar da pesquisa. Por favor, clicar em (submit) para encerrar o questionário.

Por atender aos requisitos os requisitos (Trabalhar em Manaus e ser maior de 18 anos), você pode continuar participando da pesquisa. Para continuar é necessário ler o termo de consentimento (TCLE) e, se for o caso, aceitá-lo.  Para a leitura completa do TCLE do Projeto de Pesquisa Tuberculose e Migrantes nos Países do BRICS: o caso do Brasil, acesse aqui.

 TERMO DE CONSENTIMENTO: Declaro que li o TCLE e estou ciente do uso dos meus dados sem identificação em relatórios e análises da pesquisa acima. Caso concorde ou não de participar da pesquisa clicar em (submit) para prosseguir ou encerar.

Não Concordo Concordo

Prezado(a) profissional da Atenção Primária de Manaus, este questionário faz parte de um estudo da Universidade Federal do Espírito Santo e Organização Mundial de Saúde, com aprovação da Secretaria Municipal de Saúde, para entender aspectos da atenção à saúde de migrantes internacionais na cidade. Você não precisará se identificar para respondê-lo. Garantimos o sigilo dos dados e todas as informações fornecidas serão apenas analisadas em conjunto.

**Dados da Entrevista**

1- Sexo:

Feminino Masculino

2- Raça/cor/etnia:

Branca Preta Amarela Parda Indígena Crioula Não quero responder Outra

2.1- Por favor, escreva a outra raça/cor/etnia que você se reconhece:

3-Tipo de unidade que trabalha:

UBS ESF inserida em UBS UBSF (Unidade Básica de Saúde da Família) Policlínica Ambulatório de Referência Outra

3-Qual o outro tipo de unidade  que trabalha:

4- Ocupação:

Médico Enfermeiro Técnico de Enfermagem Auxiliar de Enfermagem Outra 4.1- Qual outra ocupação?

5- Número de anos que trabalha nesta função de saúde em que trabalha realiza atividades do Plano de ação de Promoção e Atenção em Saúde aos indígenas venezuelanos?

Sim Não Não sei

6.1- Quando a unidade começou a realizar essas atividades?

Menos de 6 meses Entre 6 meses e 1 ano Entre 1 e 2 anos Mais de 2 anos

Não sei

6.2- A unidade recebeu algum recurso para a realização dessas atividades?

Sim Não Não sei

6.2.1- Que tipo de recurso?

Financeiro Insumo Profissionais Outro Não sei

6.2.2- Qual(is) outro(s) tipo(s) de recurso?

6.3- A unidade interrompeu essas atividades por algum período?

Sim Não Não sei

6.3.1- Qual o principal motivo?

Falta de recursos materiais Falta de profissionais Ausência de demanda Outro Não sei

6.3.2- Qual(is) outro(s) motivo(s)?

7- A unidade em que trabalha faz diagnóstico de pessoas com tuberculose?

Sim Não Não sei

8- A unidade em que trabalha faz tratamento de pessoas com tuberculose?

Sim Não Não sei

9- A unidade de saúde em que trabalha atende migrantes internacionais?

Sim Não Não sei

9.1- Você já atendeu algum migrante estrangeiro em sua rotina de trabalho?

Sim Não Não lembro realizou o atendimento de migrantes estrangeiros pela primeira vez?

Menos de 6 meses Entre 6 meses e 1 ano Entre 1 e 2 anos Mais de 2 anos

Não lembro

9.1.2- Entre os migrantes estrangeiros atendidos em sua rotina algum era indígena venezuelano? Sim Não Não sei Não lembro

9.1.3- Você já atendeu algum migrante estrangeiro para diagnóstico ou tratamento da tuberculose? Sim Não Não lembro

10- Você vê alguma barreira/dificuldade para o atendimento de migrantes venezuelanos nas unidades de saúde de Manaus?

Sim Não Não sei

10.1- Marque as dificuldades que identifica (marcar Idioma

quantas forem necessárias): Cultura

Acesso/seguimento

Encaminhamentos

Rede de apoio

Documentação

Outro

10.2- Qual(is) outra(s) dificuldade(s)?

11- Você recebeu algum treinamento para atender os migrantes venezuelanos?

Sim Não

12- Você gostaria de receber algum treinamento para o atendimento de migrantes internacionais? Sim Não

13- Você conhece o Plano de ação de Promoção e Atenção em saúde aos indígenas venezuelanos em Manaus? Sim Não Não sei

13.1- Como ficou sabendo do plano?

Comunicação da Secretaria de Saúde Comunicação do gestor da unidade Colega de trabalho Outro

13.1.2- Quais outros meios?

13.2- Em relação à afirmativa: O Plano de ação de Promoção e Atenção em saúde aos indígenas venezuelanos em Manaus ajuda na situação de saúde dessa população:

Concordo completamente Concordo Nem concordo nem discordo Discordo

Discordo completamente

13.3- Você recomendaria a implementação de planos semelhantes em outros municípios?

Recomendo integralmente Recomendo em parte Não sei Não recomendo

13.4- Se pudesse sugerir alguma atividade para ser realizada no âmbito do Plano de ação de Promoção e Atenção em saúde aos indígenas venezuelanos em Manaus, qual seria?

14- Em relação à afirmativa: É papel da Secretaria de Saúde promover a saúde de indígenas venezuelanos em Manaus:

Concordo completamente Concordo Nem concordo nem discordo Discordo

Discordo completamente

15- Em relação à afirmativa: É papel do profissional de saúde do SUS promover a saúde de indígenas venezuelanos em Manaus:

Concordo completamente Concordo Nem concordo nem discordo Discordo

Discordo completamente

Obrigado por ter chegado ao final da pesquisa, por favor, clique em (submit).
