# Supplementary material for: Local action plan to promote access to the health system by indigenous Venezuelans from the Warao ethnic group in Manaus, Brazil: Analysis of the plan´s development, experiences, and impact through a mixed-methods study (2020)
Source: PLoS One. 2021 Nov 15;16(11):e0259189. doi: 10.1371/journal.pone.0259189 (PMC8592448; doi:10.1371/journal.pone.0259189)
Supplement: S2 File — (DOCX) [file pone.0259189.s002.docx]

**SEMI STRUCTURED SCRIPT FOR INTERVIEW WITH MIGRANTS - MANAUS-AM -**

**PHASE 3 - Qualitative**

PIN:

Date of birth:

Race/color:

Marital status:

Number of people in the family:

Municipality of residence:

Telephone:

Nationality:

Indigenous: ( ) Yes, ( ) No

If yes, ethnicity:___________

The interview will start with the question below:

**1 – Tell us what made (Mr) (Mrs) decide to come to Brazil?**

exploration points that follow can be addressed during the answer.

- report of the fact

- support received.

**2- Tell me about (Mr) (Mrs) life before coming to Brazil?**

The following exploration points can be addressed during the answer.

- social life

- home

- income and employment

- your health conditions

- structure and social support in the region

**3 - Tell us about (Mr) (Mrs) life after arriving in Brazil?**

How is your daily life? The following exploration points can be addressed during the

answer:

-time since entering the country, where he entered and cities he lived

- job

- personal and family needs

- documental status of the immigrant:

- -regular, irregular, in regularization

- -personal documents you have

- living conditions

- your health conditions

**4 – What do (Mr) (Mrs) expect for the future**

**ROTEIRO SEMI ESTRUTURADO DE ENTREVISTA COM OS MIGRANTES- MANAUS-AM – FASE 3 - Qualitativo**

ID:

Data de nascimento:

Raça/cor:

Estado civil:

Nº de pessoas na família:

Município de residência:

Telefone:

Nacionalidade:

Indígena: ( ) Sim, ( )Não

Se sim, etnia:___________

A entrevista terá início com a pergunta abaixo:

1 – Conte o que levou o senhor (a) a decidir vir para o Brasil?

**pontos de exploração que se seguem podem ser abordados durante a resposta**

- relato do fato

- apoio recebido.

2- Me conte sobre sua vida antes de vir para o Brasil?

**Pontos de exploração que se seguem podem ser abordados durante a resposta**

- vida social

- moradia

- renda e emprego

- condições de saúde

- estrutura e suporte social na região

3 - Conte como é a sua vida após a chegada no Brasil? Como é seu cotidiano?

**pontos de exploração que se seguem podem ser abordados durante a resposta:**

-tempo decorrido desde a entrada no país, por onde entrou e cidades que morou

- emprego

- necessidades pessoais e da família

-situação documental do imigrante:

- -regular, irregular, em regularização

- -documentos pessoais que possui

- condições de moradia

- condições de saúde

- acesso ao serviço de saúde: cobertura, efetividade, adoção, implementação e sustentabilidade

- suporte social

-rede de apoio

- - expectativas

- relações sociais e de afeto

4 – O que o senhor(a) espera para o futuro?
